# Management of Thyroid Peroxidase Antibody Euthyroid Women in Pregnancy: Comparison of the American Thyroid Association and the Endocrine Society Guidelines

**DOI:** 10.1155/2013/542692

**Published:** 2013-05-12

**Authors:** L. Mehran, M. Tohidi, F. Sarvghadi, H. Delshad, A. Amouzegar, O. P. Soldin, F. Azizi

**Affiliations:** ^1^Endocrine Research Center, Research Institute for Endocrine Sciences, Shaheed Beheshti University of Medical Sciences, P.O. Box 19395-4763, Tehran, Iran; ^2^Prevention of Metabolic Disorders Research Center, Research Institute for Endocrine Sciences, Shahid Beheshti University of Medical Sciences, Tehran, Iran; ^3^Departments of Oncology, Medicine, Obstetrics, Gynecology, Pharmacology, and Physiology, Georgetown University Medical Center, Washington, DC 20057, USA

## Abstract

The presence of thyroid autoantibodies is relatively high in women of childbearing age. There is evidence that positive thyroperoxidase antibody even in euthyroid women may increase the risk of spontaneous and recurrent pregnancy loss and preterm delivery. However, the evidence is not enough to justify recommendation on the screening of pregnant women for thyroid autoantibodies or LT4 supplementation for reducing maternal or fetal complications. In this paper we reviewed the related evidence and compared the new guidelines of the American Thyroid Association and Endocrine Society with respect to the screening and management of positive thyroperoxidase antibody in euthyroid pregnant women. As there was no major contradiction or disagreement between the two guidelines, either one of two guidelines may be used by clinicians for the appropriate management of thyroid autoimmunity during pregnancy.

## 1. Introduction

Thyroid disorders especially those of autoimmune origin are common in women of reproductive age. Normal maternal physiologic changes during pregnancy lead to complex endocrine and immune modifications. Thyroid gland volume enlarges and serum levels of thyroxine (T4) and triiodothyronine increase, whereas serum TSH levels reduce [1, 2]. These modifications are related to TSH-like activity of HCG, rise in thyroxin-binding globulin (TBG) due to hyperestrogenemia and resultant altered TBG glycosylation which increases TBG half life [3], elevated glomerular filtration rate (GFR), and transplacental passage of FT4. Ten to 20% of pregnant women are positive for thyroid peroxidase (TPO) or thyroglobulin antibodies and euthyroid, of whom 16% will develop high TSH values during pregnancy and 35–50% will develop postpartum thyroiditis. The prevalence of TPOAb is even higher in women with a history of recurrent pregnancy loss, at around 17–33%, and in women with a history of subfertility, at around 10–31% [4].

TPOAb constitutes a risk factor for hypothyroidism, miscarriage, preterm delivery, perinatal death, postpartum thyroid dysfunction and impaired motor, and intellectual development in the offspring. Miscarriage, or spontaneous pregnancy loss before the 24th week of gestation, is a common pregnancy complication affecting one in five pregnant women (17–33% of gestations) [5, 6]. Many factors like maternal age, family history, environmental exposure, and maternal medical conditions may be attributed to the risk of spontaneous pregnancy loss. Preterm birth, defined as delivery before 32 weeks' gestation, occurs in 6–15% of pregnancies and accounts for 75% of prenatal deaths, physical disabilities, and adverse neurodevelopmental outcomes [7]. Therefore, preterm delivery is accompanied by a high financial, psychological, and social burden on the parents and community [8]. Potential causes have been suggested for preterm labor, including trauma, infection, cervix insufficiency, premature rupture of membrane, and medical comorbidities.

The presence of thyroid autoantibodies is relatively high in women of childbearing age. There is evidence of the potential association of thyroid autoantibodies, particularly antithyroid peroxidase antibodies (TPOAb), and increased risk of pregnancy loss and preterm delivery even in euthyroid women [9]. The prevalence of TPOAb is much higher in women with a history of recurrent miscarriage and subfertility. In particular, in iodine-sufficient populations, thyroid autoimmunity is the main cause of hypothyroidism which itself contributes to adverse obstetric and fetal outcomes even in the subclinical state [9]. Although thyroglobulin autoantibodies (TgAb) are frequently detected along with TPOAb in autoimmune hypothyroidism, TgAb are also seen in some healthy individuals without detectable TPOAb. This occurrence of TgAb is uncorrelated with abnormal TSH levels, which indicates that it may not be necessary to test for TgAb and TPOAb in the general thyroid autoimmunity screening, as TPOAb testing alone seems sufficient [10]. In those individuals with indications of autoimmune thyroid disease (AITD), however, there is evidence that testing for both TPOAb and TgAb is beneficial [11, 12]. We only refer to TPOAb in this analysis.

An association between the risk of a miscarriage and AITD has been largely confirmed in several population studies, suggesting that TPOAb presence without overt thyroid dysfunction was significantly associated with a 3- to 5-fold increase in overall miscarriage rate [9, 13–16]. The association between AITD and miscarriage, recurrent spontaneous abortions, and early pregnancy loss after *in vitro *fertilization (IVF) indicates statistically significant relationship between AITD and increased pregnancy loss and confirmed by metaanalyses that an overall relative risk of miscarriage is increased approximately 3-fold in women with AITD [9, 13–15].

Three hypotheses may underlie the association of thyroid autoimmunity with pregnancy complications: first, thyroid autoantibodies may be considered as a marker of generalized autoimmune dysfunction in the body which itself has been known to be responsible for an increased pregnancy loss; second, TPOAb euthyroid women before pregnancy are more prone to develop subclinical or overt hypothyroidism during pregnancy due to hormonal imbalance in particular in the first trimester; and third, thyroid autoimmunity is considered as one of the risk factors of infertility. Although women with TPOAb are older than those without, age can be responsible for the increased risk of pregnancy loss in TPOAb women than thyroid autoimmunity itself and its role should not be ignored [17, 18].

Given the potential association of thyroid autoimmunity and adverse pregnancy outcomes, it is of prime importance to screen pregnant women for thyroid autoimmunity and to manage thyroid antibody euthyroid women during pregnancy, when necessary.

The rapidly evolving data on the management of thyroid disorders during pregnancy have been the impetus for the development of several guidelines for the management of thyroid disorders by international thyroid associations during the past few years. Most recently, two sets of guidelines on the thyroid in pregnancy were published in the peer-reviewed literature, namely, the American Thyroid Association guidelines published in October 2011 [19] and the Endocrine Society Guidelines published in August 2012 [20]. It is the aim of this paper to compare and comment on the recommendations of these guidelines regarding thyroid autoimmunity and miscarriage or preterm delivery.

## 2. Methods

The new guidelines of the American Thyroid Association and Endocrine Society addressing thyroid dysfunction during pregnancy have been considered. These guidelines reflect the knowledge gained in the last few years and discuss and suggest recommendations related to maternal and fetal aspects of thyroid disorders during gestational period. “Spontaneous Pregnancy Loss, Preterm Delivery, and Thyroid Antibodies,” pages 1098–1100 of the guidelines of the American Thyroid Association [19] and the section on the “Autoimmune Thyroid Disease and Miscarriage,” pages 2553-2554 of the Endocrine Society Clinical Practice guidelines [20] were reviewed and their recommendations for each of the specific topics were compared. Also the last part of the section of “Hypothyroidism” in pregnancy which discusses the management of TPOAb-positive euthyroid women, identified before conception, to prevent hypothyroidism during pregnancy and reduce postpartum thyroiditis (pages 1092-1093) were compared with related section in the Endocrine Society Guidelines. The recommendations that were more complete were selected and are included in Tables 1 and 2. A recommendation of either organization that differed or was more informative was cited in the tables; otherwise the word “same” was used. In the case that a recommendation or text of one of the organizations is present in the guidelines of the other organization, the letters (R) or (T) were added, respectively.

## 3. Results

The ATA guidelines posed 9 questions and responses which resulted in 5 recommendations [19]. In the Endocrine Society Clinical Practice guidelines, one recommendation was given for the screening or management of thyroid autoimmunity in pregnancy indicating that universal screening and possible treatment cannot be recommended at this time [20]. Comparisons of the recommendations of both organizations regarding screening for TPOAb in pregnancy are given in Table 1.

As women with elevated anti-TPO antibodies are at increased risk for progression of hypothyroidism, if such women identified before conception require monitoring for hypothyroidism during pregnancy based on both guidelines, ATA recommends that serum TSH should be evaluated every 4 weeks during the first half of pregnancy and at least once between 26 and 32 m weeks gestation and Endocrine Society recommends that these women should be screened for serum TSH abnormalities before pregnancy, as well as during the first and second trimesters of pregnancy.

Neither of the organizations recommends screening for TPOAb in pregnancy to prevent spontaneous abortion and preterm delivery or even screening euthyroid women with sporadic or recurrent abortions or those women undergoing IVF procedures. However, the Endocrine Society recommends thyroid-stimulating hormone (TSH) screening in pregnant women who are positive for TPOAb. The comparison of the recommendations of the American Thyroid Association and the Endocrine Society on the treatment for thyroid antibody euthyroid women in pregnancy is presented in Table 2. None of the organizations recommends levothyroxine (LT4) therapy neither for thyroid antibody euthyroid women to prevent preterm delivery, sporadic, or recurrent miscarriage nor for women undergoing assisted reproduction technology.

Regarding the risk of postpartum thyroiditis (PPT) in TPOAb women, none of the organizations recommend LT4, iodine, or selenium treatment to prevent PPT.

## 4. Discussion

During pregnancy, there is higher risk of overt and subclinical hypothyroidism in women who are positive for thyroid antibodies before conception. Prospective studies by Glinoer et al. [21] in 1994 and Negro et al. 20 years later [22] found a progressive increase in TSH levels with progression of gestation in TPOAb euthyroid women. This is due to the comprisement in the ability of thyroid function to increase its production during gestation especially in the situation of limited thyroid hormone reserve like thyroid autoimmunity and iodine deficiency which causes imbalance between supply and increasing demand. Therefore both guidelines recommend that euthyroid women (not receiving LT4) who are positive for TPOAb require monitoring for hypothyroidism during pregnancy.

A nearly 2-fold increase in spontaneous pregnancy loss in euthyroid pregnant women tested for thyroid antibodies has been demonstrated in several studies, including a meta-analysis [9, 23, 24]. In the first prospective observational study on 552 women who were not significantly different for maternal age, thyroid hormone levels, gestational age, and the presence of cardiolipin antibodies, increased risk of miscarriage was correlated with detectable levels of thyroid autoantibodies (TPOAb, TgbAb) in the first trimester of pregnancy. The rate of miscarriage was 17% in thyroid autoantibody women compared with 8.4% for autoantibody-negative women [9]. A meta-analysis reported a clear association between the presence of thyroid antibodies and miscarriage with an odds ratio (OR) of 2.73 (95% confidence interval (CI), 2.20–3.40) in eight case-control and ten longitudinal (OR, 2.30; 95% CI, 1.80–2.95) studies. In most of these studies, serum TSH levels and the age of the participants in positive thyroid antibody group were slightly but statistically significantly higher than in the negative thyroid antibody group and so these factors may partly explain the observed results [23]. In addition, the association found in these studies does not infer causality; the mechanisms responsible are not clearly known but some reports indicate that it may probably be due to fetal resorption caused by thyroid antibodies [17, 18, 25]. In another meta-analysis of 31 studies (19 cohort and 12 case-control) involving 12,126 women, 28 showed an association between thyroid autoantibodies and miscarriage; with more than tripling in the odds of miscarriage with the presence of thyroid autoantibodies (OR: 3.90, 95%  CI = 2.48 to 6.12; *P* < 0.001) for the cohort studies and the odds ratio for miscarriage of 1.80 (95%  CI = 1.25 to 2.60; *P* = 0.002) for case-control studies [24]. Other studies [26–29], mainly have been performed on pregnant women who underwent assisted reproduction technology (ART), yielded more contradictory and less clear results.

Regarding *recurrent pregnancy loss*, most studies have found it to be associated with thyroid autoimmunity in euthyroid subjects [30–32], but the data are less robust and more contradictory than for sporadic abortions due to the other potential causes of recurrent abortions. Many of the trials did not control for other possible causes of fetal loss such as parental chromosomal anomalies, immunologic derangements, and uterine pathology [32, 33]. A retrospective case-control study (on 1588 women) showed the occurrence of higher titer of thyroid autoantibodies (TgbAb and TPOAb) in women with recurrent pregnancy loss but not in women undergoing assisted reproduction technology (ART) [31]. Another case-control study on pregnant women with the history of unexplained recurrent miscarriage revealed that the presence of thyroid autoantibodies (TPOAb and TgAb) does not convey any association with miscarriage rate in contrast with the study by Prat et al. and Iravani et al. [30] in which thyroid autoantibodies were independently associated with a higher risk of recurrent abortion.

Negro et al. conducted the first prospective randomized *interventional trials of LT4 supplementation* on 984 TPOAb-positive euthyroid pregnant women. They divided TPOAb euthyroid patients into two groups: LT4-treated and LT4-untreated groups and a TPOAb-negative control group. Treated group and control group showed a similar rate for miscarriage (3.5% and 2.4%, resp.), which was significantly lower than untreated group (13.8%) (*P* < 0.05); however the study is limited by discordance between the average time of initiating treatment (at 10.5 wks of gestation) and average time of miscarriage (8.5 wks of gestation) [22].

The meta-analysis of two randomized studies evaluated the effect of treatment with levothyroxine on miscarriage in women with normal thyroid function with thyroid autoantibodies (36% and 75% relative reductions), and when the results were pooled, there was a significant 52% relative risk reduction in miscarriages with levothyroxine (RR = 0.48, 95%  CI = 0.25  to  0.92; *P* = 0.03) [22, 24].

Regarding implementation of medical interventions for TPOAb euthyroid women with recurrent abortions, studies have demonstrated decreased rates of miscarriage with LT4 or IV-IgG treatments [34–36]. In one study, a comparison of LT4 and IV-IgG managements showed a higher rate of term delivery in the LT4-treated groups [36].

Thyroid autoimmunity may be also correlated with higher frequency of *preterm delivery* and low birth weight. Negro et al. [22] demonstrated higher rates of preterm delivery (22.4%) in TPOAb women without treatment than in TPOAb-negative women (8.2%) (*P* < 0.01; RR = 12.18; 95%  CI = 7.93–18.7) and in LT4-treated TPOAb group (7%) (*P* < 0.05; RR = 1.66; 95%  CI = 1.18–2.34). In the meta-analysis, there was a significant doubling in the odds of preterm birth with the presence of thyroid autoantibodies (2.07, 1.17  to  3.68; *P* = 0.01) [24]. One study reporting the effect of levothyroxine on preterm birth in positive TPOAb euthyroid women found a 69% relative risk reduction in preterm births with levothyroxine (RR = 0.31, 95%  CI = 0.11  to  0.90) [37].

The data are less confirmatory about the role of TPOAb in miscarriage rate in infertile patients who underwent assisted reproductive technology [26, 37, 38]. A cohort study by Bussen et al. showed a miscarriage rate of over 50% in TPOAb women who underwent a first assisted reproduction technology (ART) [26]. Toulis et al. also showed that thyroid autoantibodies in euthyroid infertile women have an association with poor pregnancy outcomes after IVF attempts [28]. A meta-analysis on 1089 subfertile euthyroid women undergoing IVF (4 prospective studies) demonstrated significantly higher risk for miscarriage in women with thyroid autoimmunity (TAI) compared with controls (RR: 1.99; 95%  CI  (1.42–1.99), *P* < 0.001) [29]. These studies found a nearly 2-fold increase risk of abortion in euthyroid women who underwent IVF [22–25], while others found no significant difference [38, 39]. Negro et al. in a retrospective study on 416 euthyroid women, who underwent ART, showed that the pregnancy and delivery rate were not affected by the presence of TPOAb [38].

There is conflicting evidence regarding the role of selenium supplementation in reducing TPOAb titers in nonpregnant women [40–44]. Only recently a single RCT by Negro et al. showed that selenium supplementation in TPOAb euthyroid women decreased the frequency of PPT and titers of TPOAb during pregnancy; however due to insufficient evidences the guidelines do not recommend selenium supplementation to reduce the PPT risk in TPOAb euthyroid women. Two other randomized clinical trials [45, 46] do not support the role of iodine or LT4 therapy in reducing PPT risk in TPOAb euthyroid women. Neither intervention decreased the prevalence of PPT. Levothyroxine reduced the degree of hypothyroidism during the hypothyroid phase of PPT and iodine increased thyroid dysfunction.

## 5. Conclusions

The current available data regarding associations between thyroid autoantibodies and spontaneous or recurrent pregnancy loss and preterm delivery is convincing. However, the reduction of these complications by treatment with LT4 supplementation is less robust. The evidence is not conclusive enough to recommend screening for thyroid autoantibodies or for the treatment of euthyroid women who are positive for thyroid autoantibodies during pregnancy. Both sets of guidelines discussed here are of high quality, and there does not seem to be contradiction or disagreement between the recommendations of the American Thyroid Association (2011) [13] and of the Endocrine Society (2012) on the screening and management of women with thyroid autoantibodies in pregnancy. Therefore, we suggest that either one of the two guidelines may be used by clinicians for appropriate and up-to-date management of thyroid autoimmunity during pregnancy.

## Figures and Tables

**Table 1:** Comparison of recommendations of the American Thyroid Association [13] and Endocrine Society [14] on the aspects of screening thyroid antibodies in pregnancy.

Topic	Recommendations	
	American Thyroid Association (2011) [13]	Endocrine Society (2012) [14]
Screening all women in the first trimester of pregnancy (to prevent spontaneous abortion or miscarriage)	Not recommended, but if identified, serum TSH should be evaluated every 4 weeks during the first half of pregnancy and at least once between 26 and 32 m weeks gestation	Not recommended, but if identified, such women should be screened for serum TSH abnormalities before pregnancy as well as during the 1st and 2nd trimesters of pregnancy
Screening euthyroid women with sporadic or recurrent abortion or in women undergoing IVF	Not recommended	Same (T&R)*
Screening for thyroid antibody in the 1st trimester of pregnancy to prevent preterm delivery	Not recommended	Same (T&R)

*The letters T and R denote that the same recommendation or conception is given in the “text” or in the “recommendation” of the guideline.

**Table 2:** Comparison of recommendations of American Thyroid Association [13] and Endocrine Society [14] on the aspects of treating thyroid antibody euthyroid women in pregnancy.

Topic	Recommendations	
	American Thyroid Association (2011) [13]	Endocrine Society (2012) [14]
LT4 or IVIG therapy to reduce the chance of sporadic or recurrent miscarriage	Not recommended	Same (T&R)*
LT4 therapy in women undergoing assisted reproduction technology	Not recommended	Same (T)
LT4 therapy in thyroid antibody euthyroid women to prevent preterm delivery	Not recommended	Same (R)
LT4, iodine, or selenium therapy to prevent postpartum thyroiditis	Not recommended	—

*The letters T and R denote that the same recommendation or concept is given in the “text” or in the “recommendation” of the guideline.

## References

[1] Lazarus JH, Kokandi A (2000). Thyroid disease in relation to pregnancy: a decade of change. *Clinical Endocrinology*.

[2] Smallridge RC, Glinoer D, Hollowell JG, Brent G (2005). Thyroid function inside and outside of pregnancy: what do we know and what don't we know?. *Thyroid*.

[3] Ain KB, Mori Y, Refetoff S (1987). Reduced clearance rate of thyroxine-binding globulin (TBG) with increased sialylation: a mechanism for estrogen-induced elevation of serum TBG concentration. *Journal of Clinical Endocrinology and Metabolism*.

[4] Glinoer D, de Nayer P, Bourdoux P (1990). Regulation of maternal thyroid during pregnancy. *The Journal of Clinical Endocrinology & Metabolism*.

[5] Ellish NJ, Saboda K, O'Connor J, Nasca PC, Stanek EJ, Boyle C (1996). A prospective study of early pregnancy loss. *Human Reproduction*.

[6] Wilcox AJ, Weinberg CR, O'Connor JF (1988). Incidence of early loss of pregnancy. *The New England Journal of Medicine*.

[7] Mathews TJ, Menacker F, MacDorman MF (2004). Infant mortality statistics from the 2002 period: linked birth/infant death data set. *National Vital Statistics Report*.

[8] Petrou S (2005). The economic consequences of preterm birth during the first 10 years of life. *An International Journal of Obstetrics and Gynaecology*.

[9] Stagnaro-Green A, Roman SH, Cobin RH, El-Harazy E, Alvarez-Marfany A, Davies TF (1990). Detection of at-risk pregnancy by means of highly sensitive assays for thyroid autoantibodies. *Journal of the American Medical Association*.

[10] Spencer C, Petrovic I, Fatemi S (2011). Current thyroglobulin autoantibody (TgAb) assays often fail to detect interfering TgAb that can result in the reporting of falsely low/undetectable serum Tg IMA values for patients with differentiated thyroid cancer. *Journal of Clinical Endocrinology and Metabolism*.

[11] Jensen E, Petersen PH, Blaabjerg O (2004). Establishment of a serum thyroid stimulating hormone (TSH) reference interval in healthy adults. The importance of environmental factors, including thyroid antibodies. *Clinical Chemistry and Laboratory Medicine*.

[12] Aras G, Gültekin SS, Küçük NO (2008). The additive clinical value of combined thyroglobulin and antithyroglobulin antibody measurements to define persistent and recurrent disease in patients with differentiated thyroid cancer. *Nuclear Medicine Communications*.

[13] Prummel MF, Wiersinga WM (2004). Thyroid autoimmunity and miscarriage. *European Journal of Endocrinology*.

[14] Glinoer D, Soto MF, Bourdoux P (1991). Pregnancy in patients with mild thyroid abnormalities: maternal and neonatal repercussions. *Journal of Clinical Endocrinology and Metabolism*.

[15] Stagnaro-Green A, Glinoer D (2004). Thyroid autoimmunity and the risk of miscarriage. *Best Practice and Research*.

[16] Poppe K, Velkeniers B, Glinoer D (2008). The role of thyroid autoimmunity in fertility and pregnancy. *Nature Clinical Practice Endocrinology and Metabolism*.

[17] Stagnaro-Green A, Abalovich M, Alexander E (2011). Guidelines of the american thyroid association for the diagnosis and management of thyroid disease during pregnancy and postpartum. *Thyroid*.

[18] De Groot L, Glinoer D, Mandel SJ (2012). Management of thyroid dysfunction during pregnancy and postpartum: an endocrine society clinical practice guideline. *The Journal of Clinical Endocrinology & Metabolism*.

[19] Matalon ST, Blank M, Levy Y (2003). The pathogenic role of anti-thyroglobulin antibody on pregnancy: evidence from an active immunization model in mice. *Human Reproduction*.

[20] Lee YL, Ng HP, Lau KS (2009). Increased fetal abortion rate in autoimmune thyroid disease is related to circulating TPO autoantibodies in an autoimmune thyroiditis animal model. *Fertility and Sterility*.

[21] Glinoer D, Riahi M, Grun JP, Kinthaert J (1994). Risk of subclinical hypothyroidism in pregnant women with asymptomatic autoimmune thyroid disorders. *Journal of Clinical Endocrinology and Metabolism*.

[22] Negro R, Formoso G, Mangieri T, Pezzarossa A, Dazzi D, Hassan H (2006). Levothyroxine treatment in euthyroid pregnant women with autoimmune thyroid disease: effects on obstetrical complications. *Journal of Clinical Endocrinology and Metabolism*.

[23] Prummel MF, Wiersinga WM (2004). Thyroid autoimmunity and miscarriage. *European Journal of Endocrinology*.

[24] Thangaratinam S, Tan A, Knox E, Kilby MD, Franklyn J, Coomarasamy A (2011). Association between thyroid autoantibodies and miscarriage and preterm birth: meta-analysis of evidence. *BMJ*.

[25] Poppe K, Glinoer D, Tournaye H (2003). Assisted reproduction and thyroid autoimmunity: an unfortunate combination?. *Journal of Clinical Endocrinology and Metabolism*.

[26] Bussen S, Steck T, Dietl J (2000). Increased prevalence of thyroid antibodies in euthyroid women with a history of recurrent in-vitro fertilization failure. *Human Reproduction*.

[27] Kim CH, Chae HD, Kang BM, Chang YS (1998). Influence of antithyroid antibodies in euthyroid women on in vitro fertilization-embryo transfer outcome. *American Journal of Reproductive Immunology*.

[28] Toulis KA, Goulis DG, Venetis CA (2010). Risk of spontaneous miscarriage in euthyroid women with thyroid autoimmunity undergoing IVF: a meta-analysis. *European Journal of Endocrinology*.

[29] Imaizumi M, Pritsker A, Kita M, Ahmad L, Unger P, Davies TF (2001). Pregnancy and murine thyroiditis: thyroglobulin immunization leads to fetal loss in specific allogeneic pregnancies. *Endocrinology*.

[30] Iravani AT, Saeedi MM, Pakravesh J, Hamidi S, Abbasi M (2008). Thyroid autoimmunity and recurrent spontaneous abortion in Iran: a case-control study. *Endocrine Practice*.

[31] Kutteh WH, Yetman DL, Carr AC, Beck LA, Scott RT (1999). Increased prevalence of antithyroid antibodies identified in women with recurrent pregnancy loss but not in women undergoing assisted reproduction. *Fertility and Sterility*.

[32] Rushworth FH, Backos M, Rai R, Chilcott IT, Baxter N, Regan L (2000). Prospective pregnancy outcome in untreated recurrent miscarriers with thyroid autoantibodies. *Human Reproduction*.

[33] Esplin MS, Branch DW, Silver R, Stagnaro-Green A (1998). Thyroid autoantibodies are not associated with recurrent pregnancy loss. *American Journal of Obstetrics and Gynecology*.

[34] Kiprov DD, Nachtigall RD, Weaver RC, Jacobson A, Main EK, Garovoy MR (1996). The use of intravenous immunoglobulin in recurrent pregnancy loss associated with combined alloimmune and autoimmune abnormalities. *American Journal of Reproductive Immunology*.

[35] Stricker RB, Steinleitner A, Bookoff CN, Weckstein LN, Winger EE (2000). Successful treatment of immunologic abortion with low-dose intravenous immunoglobulin. *Fertility and Sterility*.

[36] Vaquero E, Lazzarin N, De Carolis C, Valensise H, Moretti C, Romanini C (2000). Mild thyroid abnormalities and recurrent spontaneous abortion: diagnostic and therapeutical approach. *American Journal of Reproductive Immunology*.

[37] Negro R, Mangieri T, Coppola L (2005). Levothyroxine treatment in thyroid peroxidase antibody-positive women undergoing assisted reproduction technologies: a prospective study. *Human Reproduction*.

[38] Negro R, Formosa G, Coppola L (2007). Euthyroid women with autoimmune disease undergoing assisted reproduction technologies: the role of autoimmunity and thyroid function. *Journal of Endocrinological Investigation*.

[39] Kilic S, Tasdemir N, Yilmaz N, Yuksel B, Gul A, Batioglu S (2008). The effect of anti-thyroid antibodies on endometrial volume, embryo grade and IVF outcome. *Gynecological Endocrinology*.

[40] Gärtner R, Gasnier BCH, Dietrich JW, Krebs B, Angstwurm MWA (2002). Selenium supplementation in patients with autoimmune thyroiditis decreases thyroid peroxidase antibodies concentrations. *Journal of Clinical Endocrinology and Metabolism*.

[41] Duntas LH, Mantzou E, Koutras DA (2003). Effects of a six month treatment with selenomethionine in patients with autoimmune thyroiditis. *European Journal of Endocrinology*.

[42] Mazokopakis EE, Papadakis JA, Papadomanolaki MG (2007). Effects of 12 months treatment with lselenomethionine on serum anti-TPO levels in patients with hashimoto's thyroiditis. *Thyroid*.

[43] Karanikas G, Schuetz M, Kontur S (2008). No immunological benefit of selenium in consecutive patients with autoimmune thyroiditis. *Thyroid*.

[44] Negro R, Greco G, Mangieri T, Pezzarossa A, Dazzi D, Hassan H (2007). The influence of selenium supplementation on postpartum thyroid status in pregnant women with thyroid peroxidase autoantibodies. *Journal of Clinical Endocrinology and Metabolism*.

[45] Nøhr SB, Jørgensen A, Pedersen KM, Laurberg P (2000). Postpartum thyroid dysfunction in pregnant thyroid peroxidase antibody-positive women living in an area with mild to moderate iodine deficiency: is iodine supplementation safe?. *Journal of Clinical Endocrinology and Metabolism*.

[46] Kampe O, Jansson R, Karlsson FA (1990). Effects of L-thyroxine and iodide on the development of autoimmune postpartum thyroiditis. *Journal of Clinical Endocrinology and Metabolism*.

